# Subject-independent EEG classification of imagined swallowing: Impact of saliva vs. water paradigms

**DOI:** 10.1371/journal.pone.0353570

**Published:** 2026-07-14

**Authors:** Sevgi Gökçe Aslan, Bülent Yılmaz

**Affiliations:** 1 Biomedical Engineering Department, Inonu University, Malatya, Türkiye; 2 Electrical Engineering Department, Gulf University for Science and Technology (GUST), Hawally, Kuwait; University Putra Malaysia, MALAYSIA

## Abstract

Dysphagia poses a significant burden on global health, necessitating innovative neurorehabilitation tools. Brain-Computer Interfaces (BCIs) based on motor imagery offer a promising avenue, yet the neural differentiation between distinct swallowing paradigms remains under-explored. This study investigates the electrophysiological characteristics of imagined swallowing to establish a robust, subject-independent framework for neural decoding. We recorded EEG signals from 30 participants across two experimental paradigms: imagined saliva and imagined water swallowing. A rigorous analytical pipeline was implemented, featuring artifact removal, multidimensional feature extraction, and fold-wise statistical feature selection utilizing False Discovery Rate (FDR) correction and effect size criteria. To ensure the clinical translatability of the findings, a Leave-One-Subject-Out (LOSO) cross-validation scheme and permutation testing were employed for classification and statistical validation. Our findings demonstrate that EEG-based features can distinguish rest from imagined swallowing with near-ceiling performance (~99% accuracy), regardless of the paradigm. While the discrimination between imagined saliva and water yielded moderate accuracy (~63%), the results reveal critical insights into the inherent neural similarities of these motor imagery tasks. This study provides a statistically validated, subject-independent benchmark for decoding swallowing intentions. The high classification performance underlines the feasibility of EEG-based BCIs for dysphagia rehabilitation. While established as a proof-of-concept in healthy individuals, this framework paves the way for future neurofeedback applications in clinical populations.

## 1. Introduction

Dysphagia, which is used to describe the difficulty that occurs during the transmission of food or saliva to the stomach through the mouth, pharynx, and esophagus, significantly affects quality of life because it causes physiological, psychological, and social problems [1]. Although most patients with dysphagia do not die if not treated early, they may encounter serious medical problems such as malnutrition, dehydration, and pneumonia caused by swallowing problems [2–4]. For these reasons, early diagnosis is of vital importance to improve the living standards of patients and prevent progression that will result in death. Effective methods such as nasopharyngeal endoscopy and video fluoroscopy are used to evaluate the swallowing functionality [5,6]. However, it is not easy to apply these methods. In addition, since methods such as endoscopy can cause discomfort and stress in patients, alternative complementary approaches have been explored in research settings.

In clinical practice, Speech-Language Pathologists (SLPs) serve as the primary professionals responsible for the evaluation, diagnosis, and management of dysphagia across all etiologies and care settings [7]. The gold-standard instrumental assessments employed by SLPs include Videofluoroscopic Swallowing Study (VFSS) and Fiberoptic Endoscopic Evaluation of Swallowing (FEES), both of which provide detailed visualization of oropharyngeal swallowing physiology and are widely accepted as the reference procedures for objective dysphagia diagnosis [8,9]. These procedures allow SLPs to characterize the nature, severity, and biomechanical underpinnings of swallowing impairment, thereby guiding clinical decision-making regarding dietary modifications, compensatory strategies, and rehabilitative intervention [10]. However, both VFSS and FEES require specialized equipment and trained personnel, and carry procedural limitations: VFSS involves ionizing radiation exposure, whereas FEES requires transnasal endoscope insertion, which may cause patient discomfort and limit tolerability in certain populations [9]. These constraints underscore the need for complementary, non-invasive tools capable of supplementing SLP-led clinical evaluation—particularly for monitoring rehabilitation progress, enabling remote or repeated assessment, and supporting early screening prior to formal instrumental referral.

Recently, the investigation of brain activity has been explored as a research approach to better understand swallowing-related neural mechanisms. Many methods are used for the analysis of brain activities, such as electroencephalography (EEG) [11], functional magnetic resonance imaging (fMRI) [12], positron emission tomography (PET) [13], or magnetoencephalography (MEG) [14]. EEG is a non-invasive technique used to record electrical activity in the brain through electrodes placed on the scalp. In EEG, voltage fluctuations in the ionic current accompanying neuronal activity are measured and recorded. Electroencephalography (EEG) is one of the most widely used techniques when investigating brain activity due to its advantages, such as low cost, comfort in the patient’s position while performing the test, and high temporal resolution [15,16].

Studies with EEG are also conducted to analyze the brain activities of people who have swallowing problems. Yang et al. stated that swallowing would start with tongue movement and identified timing differences between swallowing and non-swallowing states [17]. Jestrovic et al. analyzed brain activity differences between distracted and undistracted swallowing [18]. Huckabee et al., who made the first EEG study on swallowing, investigated the role of the cerebral cortex in the motor planning and initiation of swallowing [19].

In recent years, several studies have investigated the neural basis of both actual and imagined swallowing using non-invasive neuroimaging techniques such as EEG and NIRS. These studies aim to explore how swallowing-related motor imagery (MI) can be used for assessment and rehabilitation in patients with dysphagia. Particularly, the classification of motor imagery of swallowing (MI-SW) and tongue movement (MI-TM), the evaluation of brain activation patterns, and the application of neurofeedback training have been prominent topics of investigation [20–22].

In parallel, recent advances in EEG-based motor imagery research have emphasized the use of improved feature representations, ensemble and deep learning approaches, and subject-specific modeling strategies to enhance decoding performance for subtle motor intentions [23–27]. Despite these methodological developments, EEG-based studies specifically addressing swallowing imagery remain comparatively scarce. In particular, the electrophysiological characterization of different imagined swallowing contexts has not been sufficiently explored, leaving an important gap between advances in motor imagery decoding and their application to swallowing-related rehabilitation.

Unlike prior EEG-based swallowing studies that primarily focus on distinguishing rest from swallowing-related motor imagery, the present study addresses a more fine-grained intention decoding problem. Specifically, we investigate whether imagined saliva swallowing and imagined water swallowing—two physiologically similar yet contextually distinct swallowing tasks—can be differentiated at the electrophysiological level. These two imagery conditions differ in sensory context, internal motor planning, and oral conditions, while engaging largely overlapping neural pathways, making their discrimination particularly challenging. Demonstrating separability between these imagined swallowing types extends existing work beyond coarse binary paradigms and represents a critical step toward task-specific intention decoding. Establishing this separability represents a critical step toward high-resolution, task-specific BCIs.

Building upon this foundation, the present study investigates whether different types of imagined swallowing—specifically, saliva and water swallowing—can be distinguished from rest and from each other based on EEG signals. We hypothesize that: (1) imagined swallowing tasks will produce cortical activation patterns significantly different from rest, and (2) imagined saliva swallowing and imagined water swallowing will elicit distinct neural patterns. To test these hypotheses, EEG data were recorded from 30 healthy participants using a 16-channel EEG system. Participants completed two experimental paradigms—the Induced Saliva Swallowing Paradigm and the Induced Water Swallowing Paradigm—each consisting of distinct rest and imagination phases. The acquired EEG signals were preprocessed to remove noise and artifacts, and both time-domain and frequency-domain features were extracted. A comprehensive set of statistical analyses and machine learning classifiers was then applied to evaluate feature-level differences and classification performance across conditions. The analysis utilized a robust subject-independent framework featuring Leave-One-Subject-Out (LOSO) cross-validation and False Discovery Rate (FDR) correction to evaluate classification performance across paradigms.

The results of this study contribute to a deeper understanding of how imagined swallowing engages cortical dynamics and reveal task-specific neural patterns associated with different swallowing imagery contexts. Although the findings are preliminary and based on healthy participants, they support the feasibility of EEG-based decoding of swallowing intention at multiple levels of granularity. This work provides new insights into task-specific neural dynamics, supporting the feasibility of fine-grained EEG decoding for future neurofeedback and BCI applications.

## 2. Materials and methods

### 2.1 Participants

We conducted experiments with 30 people (15 women) aged between 19 and 56, with a mean age of 30 and a standard deviation of 12. The participants did not have swallowing problems or any related diseases. Ethical approval was obtained from the Erciyes University Ethics Committee (approval number 2023/461, July 12, 2023). All procedures performed in studies involving human participants were conducted following the ethical standards of the institutional research committee. All participants provided written informed consent before their inclusion in the study. The recruitment period for this study began on July 15, 2023, and ended on July 15, 2025.

### 2.2 Materials

**Fig 1:**
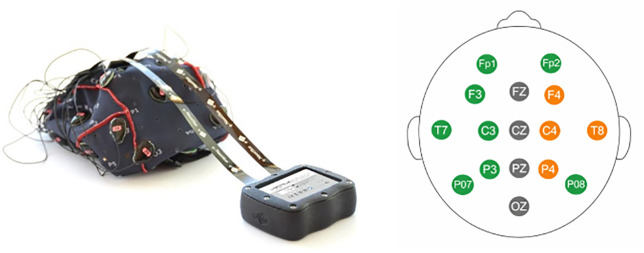
Left panel: Nautilus research-grade wearable EEG headset [28], and right panel: international 10-20 electrode placement system.

In our experiments, we used a 16-channel Nautilus research-grade wearable EEG headset device (g.tec medical engineering, Schiedlberg, Austria) to obtain EEG signals (Fig 1). Thanks to the wearable nature of the headset, the headset with dry electrodes was comfortably placed on the head of the participant. EEG electrodes were positioned according to the international 10–20 system. A sampling frequency of 500 Hz was used for the signal. The positions of the electrodes placed on the skull of the subjects can be seen in Fig 1. We recorded the EEG data in the 0.5–200 Hz range via MATLAB Simulink using the Windows operating system.

#### 2.2.1 Experimental procedure.

In this study, we abstained from attaching any devices or sensors to the neck or other body parts to detect swallowing. Instead, we conducted the entire study based on EEG signals. Our focus extended beyond the swallowing event to analyze brain activities associated with swallowing. We incorporated additional scenarios, such as periods of rest before swallowing and the mental imagery of swallowing, into our experimental protocols. During the experiment, participants were instructed to follow the statements displayed on the screen, to keep their eyes open and maintain visual focus, and were explicitly advised to avoid any additional physical movements. The study employed two approved experimental paradigms. Consequently, our analyses were conducted through two distinct experiments. This comprehensive approach allowed us to gain insights into the act of swallowing and the broader spectrum of factors influencing brain activities. By exploring various conditions, we aimed to provide a better understanding of the neural processes associated with swallowing.

***Paradigm 1: Induced saliva swallowing***

**Fig 2:**
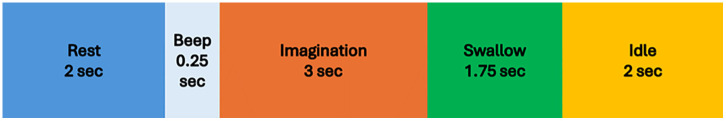
Saliva swallowing test procedure with corresponding timing information. Only the rest (0–2 s) and imagined swallowing (2.25–5.25 s) phases were analyzed in this study. The actual swallowing phase was excluded from EEG analysis.

In this experimental paradigm, participants were instructed to engage in the mental process of swallowing following a period of rest, and subsequently, to simulate swallowing without the ingestion of any food or drink. This specific trial, referred to as the ‘saliva swallowing’ experiment, aimed to investigate the impact of simulated swallowing without oral intake on EEG signals. The experiment encompassed distinct phases, including resting, imagining, and actual swallowing. During the resting phase, participants focused on a plus symbol displayed on the screen for 2 seconds. After the auditory beep cue, when the word “imagination” appeared on the screen, participants were instructed to internally simulate the act of swallowing saliva without making any overt motor movements. They were explicitly told to keep their eyes open, maintain visual fixation on the screen, and refrain from any physical motion throughout the imagination period. In the final stage of the swallowing component, participants observed the word ‘swallow’ on the computer screen and executed the physical act of saliva swallowing. Each trial within this experiment lasted 9 seconds, with a total of 15 trials amounting to 135 seconds. Figure 2 provides a graphical representation of a 9-second segment from the saliva swallowing paradigm.

***Paradigm 2: Induced water swallowing***

In this experimental paradigm, termed ‘induced water swallowing,’ we aimed to discern the resulting variances in EEG signals by instructing participants to imagine swallowing a sip of water drawn into the mouth. This experiment encompassed four stages: drawing water from a bottle using a straw, resting, imagining, and actual swallowing. Participants were provided with a bottle of water and a straw and instructed to follow the cues displayed on the screen. A key distinction from the previous experiment lay in the presence of water in the subject’s mouth during the imagination phase.

The initial stage prompted participants to draw water from the bottle using the straw upon seeing the text ‘draw water’ on the screen. Subsequently, a plus sign appeared for 2 seconds, during which participants engaged in the resting phase by focusing on the symbol. In the third stage, the word ‘imagination’ was displayed on the screen, instructing participants to mentally simulate swallowing water in their mouths for a duration of 3 seconds. The final stage entailed participants physically swallowing the water.

**Fig 3:**
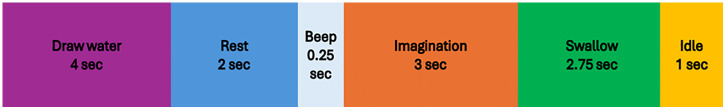
Induced water swallowing test procedure with corresponding timing information. Only the rest (4–6 s) and imagined swallowing (6.25–9.25 s) phases were included in the EEG analysis. The water intake and actual swallowing phases were excluded to avoid contamination from motor artifacts.

Each segment of this experiment lasted 13 seconds, with the entire process repeated 15 times, amounting to a total duration of 195 seconds. Figure 3 illustrates a 13-second excerpt from this experiment. Since this paradigm involved the physical action of drawing water into the mouth via a straw, the total duration of each trial was naturally longer compared to the saliva swallowing experiment. However, only the rest and imagination phases were included in the EEG analysis, while the water intake and actual swallowing stages were explicitly excluded to prevent contamination from motor-related activity. Therefore, the EEG features extracted in this experiment, as in the saliva swallowing paradigm, are based solely on internal cognitive processes. This methodological consistency ensures that comparisons between the two experimental paradigms (saliva vs. water imagery) remain valid and scientifically sound, as both rely on equivalent rest and mental imagery conditions free from physical movement artifacts.

### 2.3 Data analysis

All EEG data processing steps, including preprocessing and feature extraction, were performed using MATLAB scripts based on predefined task intervals. Preprocessing and feature extraction were applied independently to each subject using predefined task intervals, whereas all data-dependent classification steps, including statistical feature selection, were performed only within each LOSO training fold. Since no manual selection or visual inspection of EEG segments was applied, inter- and intra-rater reliability assessments were not applicable.

For each fold i (i = 1,..., 30), the following steps were executed in strict sequence: (1) The held-out subject i was designated as the test set; data from the remaining 29 subjects constituted the training set. (2) Missing or non-finite feature values were imputed using the median of each feature computed exclusively from the training set. (3) Subject-level averaging was performed: for each training subject, the mean feature vector was computed separately for the rest condition (15 trials) and the imagination condition (15 trials), yielding n = 29 paired subject-level observations per fold. (4) Statistical feature selection was applied to these 29 paired observations using FDR-corrected paired tests (see Section 2.5); only features surviving both the significance and effect-size thresholds were retained. This step used only training data. (5) Z-score normalization was applied using the mean and standard deviation of the training set computed over the selected feature subset; these parameters were then applied identically to the test subject’s data. This order ensures that normalization is performed on the same feature space used for classification, avoiding any information leakage from redundant features. (6) Classifiers were trained on the selected and normalized features using all training subjects’ trial-level data. (7) The trained classifier was applied to the held-out test subject’s data, preprocessed using the training-set normalization parameters, to obtain the final fold-level performance estimate. The test subject’s data did not influence any of steps 2–6, ensuring a fully leakage-free evaluation. Note that subject-level averaging was applied exclusively within the statistical feature selection step (step 3–4); classifier training and evaluation (steps 6–7) were performed on trial-level data to preserve the full statistical power of the training set.

Regarding the permutation test, feature selection within each permutation fold was performed using a paired t-test rather than the full normality-gated procedure (Shapiro-Wilk/Lilliefors followed by paired t-test or Wilcoxon signed-rank test) employed in the main analysis. This simplification was adopted to maintain computational feasibility across 500 permutation iterations while preserving the within-subject paired structure of the comparison. As the permutation test is used solely to generate a null distribution against which real performance is compared, this approximation does not affect the validity of the resulting p-values.

#### 2.3.1 Filtering.

EEG data provides essential information in neurological studies. However, this data is often contaminated with noise and unwanted components. Therefore, it is crucial to clean EEG data and remove unwanted signals before conducting data analysis. In this section, we will examine in detail three different filtering methods used for cleaning EEG data acquired during the experiments.

***Empirical Mode Decomposition (EMD) method***

Empirical Mode Decomposition (EMD) is a nonlinear, adaptive, and data-driven signal processing technique designed to analyze non-stationary and complex signals such as electroencephalography (EEG). Unlike traditional filtering methods that rely on predefined basis functions, EMD decomposes a signal directly based on its intrinsic oscillatory characteristics. This property makes EMD particularly suitable for EEG analysis, where neural signals exhibit time-varying frequency and amplitude components.

In EMD, an EEG signal is decomposed into a finite number of intrinsic mode functions (IMFs), each representing oscillatory modes at different frequency scales. Lower-order IMFs typically capture high-frequency components, which are often associated with noise and artifacts, whereas higher-order IMFs reflect slower oscillatory patterns related to meaningful neural activity. By selectively retaining appropriate IMFs, EMD enables effective noise reduction while preserving physiologically relevant information.

In this study, EMD was applied independently to each EEG channel as a preprocessing step to enhance signal quality prior to feature extraction. Each EEG signal was decomposed into ten intrinsic mode functions.

**Fig 4:**
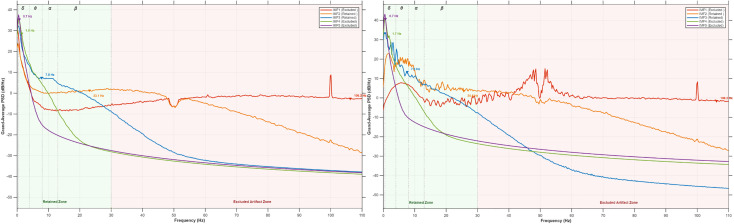
Grand-average PSD-based selection of intrinsic mode functions (IMFs) at channel Cz across 30 subjects.

To ensure physiologically meaningful reconstruction, an empirical IMF selection procedure was performed across all 30 subjects for both experimental paradigms. Grand-average power spectral density (PSD) and dominant frequency analyses at Channel Cz were used to characterize the spectral properties of each intrinsic mode function (IMF). Consistent spectral patterns were observed across both experiments, confirming the robustness of the decomposition (see Fig 4).

In both Experiment 1 and Experiment 2, IMF1 consistently exhibited very high dominant frequencies (106.24 ± 44.76 Hz and 106.15 ± 40.93 Hz, respectively) together with dominant high-frequency energy (>30 Hz: 10.1% and 83.4%), indicating strong contamination from EMG and non-neural artifacts. Similarly, IMF4 and IMF5 were dominated by slow-wave and sub-delta activity with dominant frequencies below 2 Hz, reflecting physiological drift and non-task-related fluctuations, making them unsuitable for motor imagery analysis.

In contrast, IMF2 and IMF3 consistently demonstrated physiologically relevant spectral characteristics across both experiments. IMF2 showed dominant frequencies in the beta range (23.09 ± 13.09 Hz and 25.62 ± 10.35 Hz), while IMF3 consistently reflected alpha/mu band activity (7.84 ± 4.85 Hz and 7.60 ± 4.75 Hz). Their PSD distributions confirmed that the majority of signal energy was concentrated within the 0.5–30 Hz range, which is known to contain task-relevant motor imagery oscillations.

Based on this convergent spectral evidence across both experimental paradigms, IMF2 and IMF3 were selected for subsequent analysis, as they consistently captured meaningful neural oscillations while effectively excluding both high-frequency artifacts and low-frequency drift components.

***Band-pass filtering (0.5–40 Hz)***

Band-pass filtering was applied to EEG data to preserve a specific frequency range (0.5–40 Hz) using the EEGlab toolbox [29]. EEGlab is a powerful open-source tool for processing EEG data. Through this method, we retained the desired frequency components in EEG data while filtering out noise and unwanted components.

***Independent Component Analysis (ICA)***

Using the EEGlab toolbox, Independent Component Analysis (ICA) was employed to clean EEG data. ICA enables the separation and isolation of different sources within EEG data. Our EEG signal contained signals such as muscle activity and eye movements. ICA was applied to the multichannel EEG data to decompose the signals into statistically independent components, rather than being applied separately to each channel. Artifact-related components were identified and removed, resulting in cleaner EEG signals for subsequent analysis. This approach is consistent with standard EEG preprocessing practices.

### 2.4 Feature extraction

We extracted 30 frequency- and time-domain features from each EEG channel corresponding to the rest and imagination phases of different swallowing paradigms. These features were carefully selected based on their established effectiveness in EEG analysis and motor imagery studies, particularly for capturing neurophysiological complexity and cognitive workload. The feature extraction approaches employed included the Ratio of Band Power (Alpha to Beta), Band Power Gamma, Band Power Beta, Band Power Alpha, Band Power Theta, Band Power Delta, Hjorth Activity, Hjorth Mobility, Hjorth Complexity, Skewness, Kurtosis, First Difference, Normalized First Difference, Second Difference, Normalized Second Difference, Mean Curve Length, Mean Energy, Mean Teager Energy, Log Root Sum of Sequential Variation, Tsallis Entropy, Shannon Entropy, Log Energy Entropy, Renyi Entropy, Arithmetic Mean, Standard Deviation, Variance, Median Value, Auto-Regressive Model, Maximum Value, and Minimum Value [30–33].

These 30 features were extracted from the 16 channels for each imagination and rest segment. Consequently, for each segment, we obtained a total of 480 features (30 x 16). This feature selection strategy was designed to enhance the ability to detect neurophysiological distinctions between conditions and to improve classification performance.

### 2.5 Statistical analysis

Statistical analyses were conducted to identify EEG features that significantly differed between the rest and imagined swallowing conditions and to control the dimensionality of the extracted feature space. Since the experimental design involved repeated measurements within the same subjects, all statistical comparisons were performed at the subject level to avoid inflated sample sizes due to trial-level dependencies.

For each training subject within the leave-one-subject-out (LOSO) cross-validation framework, feature values were first summarized separately for the rest and imagination conditions by computing the mean value across trials. These subject-level summaries were then used to perform paired statistical comparisons between the two conditions for each feature. This procedure yielded n = 29 paired observations per fold (one per training subject), so that each subject contributed exactly one data point to the statistical comparison. Treating individual trials as independent observations would artificially inflate the sample size to n = 435 (29 subjects × 15 trials), producing misleadingly low p-values; subject-level averaging prevents this pseudo-replication. Thus, all statistical inferences were based on subject-level paired observations rather than pooled trial-level data. All normalization parameters (mean and standard deviation) were computed using only the training data within each LOSO fold and subsequently applied to the held-out test subject.

To determine whether the paired feature differences followed a normal distribution, a normality test was applied to the within-subject difference vector d = y − x, where x and y denote the subject-level means for the rest and imagination conditions, respectively, across the n = 29 training subjects in each fold. If the difference vector d was normally distributed (assessed via Lilliefors test), a one-sample paired t-test was applied directly to d (equivalent to ttest(d) in MATLAB), yielding Cohen’s d as the effect size. If normality was violated, the Wilcoxon signed-rank test was applied to the paired observations (x, y) as a non-parametric within-subject alternative. In both cases, the test operated on within-subject differences, not on independent group comparisons. This within-subject paired design ensured that the statistical testing procedure appropriately reflected the repeated-measures nature of the experiment and avoided inflated degrees of freedom that would arise from treating individual trials as independent observations.

Given the large number of extracted features, multiple-comparison correction was applied using the Benjamini–Hochberg false discovery rate (FDR) procedure with a significance level of α = 0.05. Features that passed the FDR-corrected significance threshold were considered statistically relevant.

In addition to statistical significance, effect size measures were computed to assess the practical relevance of the observed differences. For normally distributed paired differences, Cohen’s d was calculated as the standardized mean difference. For non-normal paired comparisons, the Wilcoxon effect size r was computed using the standardized test statistic. To ensure that only features with meaningful discriminative power were retained, effect-size thresholds were applied (|d| ≥ 0.5 for Cohen’s d and |r| ≥ 0.3 for Wilcoxon r).

Only features satisfying both the FDR-corrected significance criterion and the effect-size threshold were selected for the subsequent classification stage. Importantly, this feature selection procedure was performed independently within each LOSO training fold using only training subjects, ensuring that no information from the held-out test subject influenced the selection process. If no features satisfied both criteria within a particular LOSO fold, a fallback strategy was applied in which a small subset of features with the lowest p-values was selected to maintain classifier stability.

To further evaluate the statistical validity of the classification results, a permutation test was performed. In this procedure, class labels were randomly shuffled across trials within each subject, and the entire classification pipeline was repeated 500 times to generate a null distribution of classification accuracies. The empirical p-value was calculated as the proportion of permutation accuracies that were equal to or greater than the real classification accuracy. To further rule out the possibility that trial-order-related temporal drift contributed to classification performance, a temporal cue analysis was performed. For each subject, the Spearman rank correlation between trial number (1–15) and epoch-level RMS amplitude was computed separately for rest and imagination epochs. Multiple-comparison correction was applied using the Benjamini–Hochberg FDR procedure (α = 0.05). This procedure allowed us to assess whether the observed decoding performance was significantly above chance level. All statistical analyses and classification procedures were implemented in MATLAB using custom scripts.

### 2.6 Classification

In this study, various classification methods were employed to discriminate between the rest and imagination phases in the Induced Saliva Swallowing and Induced Water Swallowing experiments using the Leave-One-Subject-Out Cross-Validation (LOSO-CV) method (30-fold). Additionally, classification was performed on the imagination data from these experiments to facilitate comparative analysis and gain insights into the distinctive features associated with each paradigm.

During the comparison of classification approaches, careful selection of hyperparameters is important for ensuring a fair and consistent evaluation across models. In this study, instead of performing extensive hyperparameter optimization, fixed and commonly used hyperparameter settings were employed for each classifier. This approach was adopted to reduce the risk of overfitting and to avoid additional model-selection bias within the leave-one-subject-out (LOSO) cross-validation framework.

These predefined settings were selected based on standard implementations and commonly reported configurations in the literature, ensuring comparability across classifiers.

By including multiple classifiers, we aimed to evaluate which algorithms perform best under the specific characteristics of our dataset while minimizing model-specific bias. This comparative approach also facilitates reproducibility and provides guidance for future studies in EEG-based BCI applications for dysphagia rehabilitation.

Eight machine learning classifiers (KNN, SVM, Decision Tree, Naïve Bayes, Random Forest, AdaBoost, Bagging, and Kernel Classifier) were evaluated under the LOSO framework. The corresponding hyperparameter settings are summarized in Table 1.

**Table 1:** Hyperparameters used for machine learning classifiers.

Classifier	Key hyperparameters
K-Nearest Neighbors (KNN)	k = 3; distance metric = Euclidean
Support Vector Machine(SVM)	Kernel = linear (default); C = default
Decision Tree	Split criterion = Gini; maximum depth = default
Naive Bayes	Gaussian distribution (default)
AdaBoost	AdaBoostM1; 100 weak learners; base learner = decision tree
Bagging	100 base learners; base classifier = decision tree
Random Forest	100 trees; feature sampling = default (TreeBagger)
Kernel Classifier	Kernel type = default (fitckernel)

Performance was evaluated using Accuracy, Precision, Recall, and F1-score calculated from True Positive (tp), True Negative (tn), False Positive (fp), and False Negative (fn) values, according to Equations (1)–(4).


$$\begin{array}{c} Precision=\frac{\text{tp}}{\text{tp}+\text{fp}} \end{array}$$ (1)

$$\begin{array}{c} Recall=\frac{\text{tp}}{\text{tp}+\text{fn}} \end{array}$$ (2)

$$\begin{array}{c} Accuracy=\frac{\text{tp}+\text{tn}}{\text{tp}+\text{tn}+\text{fn}+\text{fp}} \end{array}$$ (3)

$$\begin{array}{c} F-measure=\frac{\text{2}\times \text{Precision}\times \text{Recall}}{\text{Precision}+\text{Recall}} \end{array}$$ (4)

The results of these analyses are presented in the following sections, allowing a comprehensive understanding of the effectiveness of each classification approach in distinguishing between rest and imagination states across the two experimental paradigms and between imagination conditions in the two experiments.

## 3. Results

Classification analyses were performed to evaluate the discriminability of EEG features across three conditions: (i) rest versus imagined saliva swallowing in Paradigm 1, (ii) rest versus imagined water swallowing in Paradigm 2, and (iii) imagined saliva versus imagined water swallowing in the cross-paradigm setting. Performance was assessed using eight machine learning classifiers under leave-one-subject-out cross-validation, considering both the full feature set and the statistically selected feature subset. Overall, the rest-versus-imagery classifications in both paradigms yielded very high accuracies, whereas cross-paradigm decoding between imagined saliva and imagined water swallowing was substantially more challenging.

### 3.1 Classification performance for Paradigm 1 (rest vs. imagined saliva swallowing)

The classification performance for Paradigm 1, which aimed to discriminate between rest and imagined saliva swallowing conditions, is summarized in Table 2. Overall, very high classification accuracies were obtained for most machine learning models, indicating that the extracted EEG features effectively captured the neural patterns associated with imagined swallowing.

**Table 2:** Classification performance for rest vs imagined swallowing using all and selected extracted features under LOSO cross-validation for Paradigm 1.

Model	Paradigm 1 (All)				Paradigm 1 (Selected)			
	Accuracy (%)	Precision (%)	Recall (%)	F1 (%)	Accuracy (%)	Precision (%)	Recall (%)	F1 (%)
**KNN**	96.22	98.22	94.95	96.42	97.44	99.56	96.01	97.63
**SVM**	99.89	99.78	100.00	99.89	99.44	99.56	99.38	99.45
**Decision Tree**	98.00	98.00	98.40	98.06	98.11	98.22	98.38	98.18
**Naive Bayes**	55.22	16.00	49.66	20.63	67.00	39.11	81.46	47.77
**Random Forest**	99.22	99.33	99.19	99.22	99.44	99.33	99.58	99.43
**AdaBoost**	99.56	99.78	99.38	99.56	99.56	99.78	99.36	99.56
**Bagging**	99.33	99.33	99.40	99.33	99.33	99.33	99.38	99.32
**Kernel**	48.78	49.33	48.81	48.29	50.11	48.89	50.85	49.24

Using all extracted features, several classifiers achieved near-perfect performance. Among them, the Support Vector Machine (SVM) classifier yielded the highest accuracy of 99.89%, accompanied by precision, recall, and F1-score values of 99.78%, 100.00%, and 99.89%, respectively. Ensemble-based classifiers also demonstrated excellent performance. AdaBoost achieved an accuracy of 99.56%, while Bagging and Random Forest reached 99.33% and 99.22%, respectively, with highly balanced precision and recall values. These results indicate robust and reliable classification performance across subjects.

Traditional machine learning approaches also produced strong results. K-Nearest Neighbors (KNN) achieved an accuracy of 96.22%, while the Decision Tree classifier reached 98.00%, further confirming the separability of rest and imagined saliva swallowing states within the extracted EEG feature space.

In contrast, the Naïve Bayes and Kernel classifiers exhibited substantially lower performance. Naïve Bayes achieved an accuracy of 55.22% with a notably low F1-score of 20.63%, while the Kernel classifier obtained an accuracy of 48.78%. These findings suggest that these classifiers were less capable of modeling the complex and non-linear distribution of the EEG feature space.

When the statistically selected feature subset was used, classification performance remained stable and, for some classifiers, improved slightly. KNN accuracy increased from 96.22% to 97.44%, while Random Forest improved from 99.22% to 99.44%. Decision Tree performance also showed a marginal increase from 98.00% to 98.11%. AdaBoost maintained the highest overall accuracy of 99.56%, demonstrating the robustness of the selected feature subset. Although SVM accuracy decreased slightly to 99.44%, its performance remained exceptionally high. Furthermore, Naïve Bayes showed the largest improvement, with accuracy increasing from 55.22% to 67.00% and F1-score improving from 20.63% to 47.77%, indicating that feature selection helped mitigate the effects of redundant or noisy features.

Overall, the results demonstrate that the EEG features extracted from Paradigm 1 enable highly accurate discrimination between rest and imagined saliva swallowing conditions. The consistently high performance of SVM and ensemble learning methods, particularly AdaBoost, Random Forest, and Bagging, highlights their effectiveness for EEG-based imagined swallowing classification. Additionally, the feature selection procedure successfully reduced feature dimensionality while preserving or slightly improving classification performance.

### 3.2 Classification performance for Paradigm 2 (rest vs. imagined water swallowing)

The classification results for Paradigm 2, which aimed to distinguish between the resting state and imagined water swallowing conditions, are presented in Table 3. Overall, most classifiers achieved high performance, indicating that the extracted EEG features contain strong discriminative information between the two conditions.

**Table 3:** Classification performance for rest vs imagined swallowing using all and selected extracted features under LOSO cross-validation for Paradigm 2.

Model	Paradigm 2 (All)				Paradigm 2 (Selected)			
	Accuracy (%)	Precision (%)	Recall (%)	F1 (%)	Accuracy (%)	Precision (%)	Recall (%)	F1 (%)
**KNN**	95.22	98.67	93.58	95.74	96.11	99.11	94.71	96.57
**SVM**	99.33	99.33	99.37	99.33	98.22	97.56	99.02	98.15
**Decision Tree**	98.00	98.44	97.75	98.01	97.89	97.56	98.34	97.85
**Naïve Bayes**	57.56	22.22	58.78	27.71	56.67	19.56	58.45	25.42
**Random Forest**	98.89	99.33	98.72	98.96	99.00	99.33	98.88	99.04
**AdaBoost**	99.11	100.00	98.43	99.17	99.00	99.78	98.49	99.07
**Bagging**	99.00	99.56	98.67	99.06	98.67	99.11	98.57	98.73
**Kernel**	50.67	51.11	50.86	50.70	49.00	47.56	48.47	47.70

Using the full feature set, several classifiers demonstrated near-ceiling performance. Among them, the Support Vector Machine (SVM) achieved the highest accuracy of 99.33%, with precision, recall, and F1-score values of 99.33%, 99.37%, and 99.33%, respectively. AdaBoost also showed very strong performance with an accuracy of 99.11%, followed closely by Bagging (99.00%) and Random Forest (98.89%). These results indicate that both margin-based and ensemble learning methods are highly effective in capturing discriminative EEG patterns associated with imagined water swallowing.

Traditional machine learning methods also produced competitive results. The K-Nearest Neighbors (KNN) classifier achieved an accuracy of 95.22%, while the Decision Tree classifier reached 98.00%, further supporting the separability of the two conditions in the feature space. In contrast, Naïve Bayes and Kernel-based classifiers showed substantially lower performance, with accuracies of 57.56% and 50.67%, respectively, suggesting limited ability to model the complex and likely non-Gaussian distribution of EEG-derived features.

When the statistically selected feature subset was used, classification performance remained largely consistent across most classifiers. Random Forest and AdaBoost achieved the highest accuracies in this setting, both reaching 99.00%. Bagging and SVM obtained accuracies of 98.67% and 98.22%, respectively. KNN showed a slight improvement in precision and overall accuracy, reaching 96.11%. Minor variations were observed across classifiers; however, the overall performance trend remained stable, indicating that the feature selection process preserved the most informative EEG features while reducing dimensionality.

Overall, the findings of Paradigm 2 demonstrate that imagined water swallowing can be reliably distinguished from the resting state with very high accuracy. In particular, SVM and ensemble-based methods consistently showed superior performance, confirming the robustness and discriminative power of the extracted EEG feature representation.

### 3.3 Cross-paradigm decoding (imagined saliva vs. imagined water swallowing)

The cross-paradigm decoding results obtained for the classification of imagined saliva swallowing and imagined water swallowing are summarized in Table 4. In contrast to the rest-versus-imagery classification tasks presented in Paradigms 1 and 2, substantially lower classification performances were observed for this task, indicating a greater degree of similarity between the neural representations of the two imagined swallowing conditions.

**Table 4:** Cross-Paradigm decoding performance (imagined saliva vs imagined water) using all features and statistically selected features under LOSO cross-validation.

Model	All features				Selected features			
	Accuracy (%)	Precision (%)	Recall (%)	F1 (%)	Accuracy (%)	Precision (%)	Recall (%)	F1 (%)
**KNN**	53.89	74.67	53.84	60.91	50.22	53.33	50.50	49.25
**SVM**	62.67	65.11	64.62	59.40	51.11	84.22	48.56	60.24
**Decision Tree**	61.67	61.56	62.41	58.86	49.89	53.11	51.90	49.23
**Naïve Bayes**	56.89	87.56	57.43	66.55	52.89	91.33	50.27	64.46
**Random Forest**	60.22	60.67	64.42	54.83	48.78	50.67	51.44	46.79
**AdaBoost**	63.56	64.44	61.06	60.38	55.22	66.22	55.04	56.30
**Bagging**	61.78	62.22	60.37	56.37	48.67	50.89	51.35	47.02
**Kernel**	51.33	48.44	51.58	49.34	54.78	74.44	51.90	58.95

Using all extracted features, the highest classification accuracy was achieved by the AdaBoost classifier (63.56%), followed by SVM (62.67%), Bagging (61.78%), and Decision Tree (61.67%). Random Forest also produced comparable performance with an accuracy of 60.22%. KNN and Naïve Bayes achieved accuracies of 53.89% and 56.89%, respectively, while the Kernel classifier yielded an accuracy of 51.33%, close to chance level. Although ensemble-based classifiers generally outperformed the other methods, the overall classification performance remained substantially lower than that observed in the rest-versus-imagery paradigms.

The relatively modest accuracies suggest that the EEG patterns associated with imagined saliva swallowing and imagined water swallowing share considerable overlap. This finding indicates that the two motor imagery tasks recruit highly similar neural mechanisms, making their discrimination considerably more challenging than separating either imagery condition from rest.

When the statistically selected feature subset was used, classification performance generally decreased across most classifiers. AdaBoost again achieved the highest accuracy (55.22%), followed by the Kernel classifier (54.78%) and Naïve Bayes (52.89%). More pronounced performance reductions were observed for SVM, Decision Tree, Random Forest, and Bagging, whose accuracies decreased to approximately chance level. These results suggest that the discriminative information required to distinguish between the two imagined swallowing tasks is distributed across a broader feature space and may not be adequately preserved by the feature selection procedure.

Notably, Naïve Bayes achieved exceptionally high precision values in both the all-feature and selected-feature analyses (87.56% and 91.33%, respectively), despite relatively modest accuracy and recall values. Similarly, the selected-feature SVM and Kernel classifiers produced high precision values of 84.22% and 74.44%, respectively. These findings indicate that some classifiers generated highly confident predictions for a subset of samples while exhibiting limited generalization across the complete dataset.

Overall, the cross-paradigm results demonstrate that, although imagined swallowing conditions can be reliably distinguished from rest, differentiating between imagined saliva swallowing and imagined water swallowing remains substantially more difficult. The moderate classification accuracies observed across all models suggest the presence of highly overlapping neural activity patterns underlying the two swallowing imagery tasks.

### 3.4 Comparison of classifier performance

**Fig 5:**
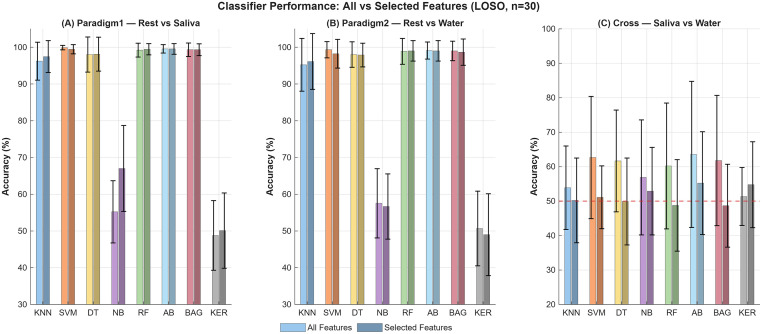
LOSO (n = 30) classification accuracies for all vs. selected features across classifiers. **(A)** Paradigm 1: Rest vs. imagined saliva swallowing. **(B)** Paradigm 2: Rest vs. imagined water swallowing. **(C)** Cross-paradigm classification: imagined saliva vs. imagined water swallowing. Error bars represent the standard deviation across subjects.

A comparison of the classification performances obtained using all features and the statistically selected feature subset across different classifiers is presented in Fig 5.

As shown in Fig 5(A) and Fig 5(B), most classifiers achieved very high accuracies for both Paradigm 1 (rest vs. imagined saliva swallowing) and Paradigm 2 (rest vs. imagined water swallowing). SVM and ensemble-based classifiers, particularly Random Forest, AdaBoost, and Bagging, consistently demonstrated the strongest performance, with accuracies generally ranging from 98% to 100%. In contrast, Naïve Bayes and Kernel classifiers exhibited substantially lower accuracies and greater variability than the other methods.

The effect of feature selection differed across paradigms. For Paradigm 1, the selected feature subset maintained or slightly improved performance for classifiers, particularly Naïve Bayes, while preserving the near-ceiling performance of the best-performing models. For Paradigm 2, classification accuracies remained largely stable after feature selection, with only minor increases or decreases depending on the classifier. These findings suggest that the selected feature subset retained most of the discriminative information required for rest-versus-imagery classification while reducing feature dimensionality.

In contrast, markedly lower accuracies and larger standard deviations were observed for the cross-paradigm classification task shown in Fig 5(C). Using all features, the highest accuracy was achieved by AdaBoost. After feature selection, performance decreased for most classifiers, with the highest accuracy dropping to AdaBoost. The larger error bars observed in this paradigm indicate considerable inter-subject variability and suggest that the neural activity patterns associated with imagined saliva swallowing and imagined water swallowing are highly overlapping. Furthermore, the reduction in performance following feature selection implies that the discriminative information required for cross-paradigm decoding is distributed across a broader feature space and may not be adequately captured by a reduced feature set.

Overall, the results indicate that SVM and ensemble learning approaches provide the most reliable and stable performance for rest-versus-imagery classification. Moreover, while the proposed feature selection strategy effectively preserves classification performance in Paradigms 1 and 2, it appears less suitable for the more challenging cross-paradigm decoding task, where a larger set of features may be necessary to capture subtle differences between imagined saliva and imagined water swallowing.

### 3.5 Subject-level classification performance

**Fig 6:**
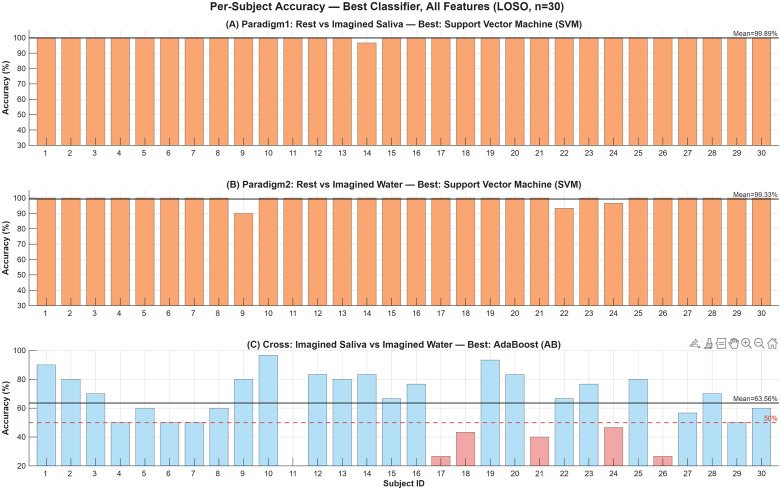
Subject-wise classification accuracies obtained using the best-performing classifiers with the full feature set under LOSO-CV. **(A)** Paradigm 1: Rest versus imagined saliva swallowing, where the best-performing classifier was Support Vector Machine (SVM). **(B)** Paradigm 2: Rest versus imagined water swallowing, where the best-performing classifier was AdaBoost (AB). **(C)** Cross-paradigm classification of imagined saliva versus imagined water swallowing, where the best-performing classifier was AdaBoost (AB).

**Fig 7:**
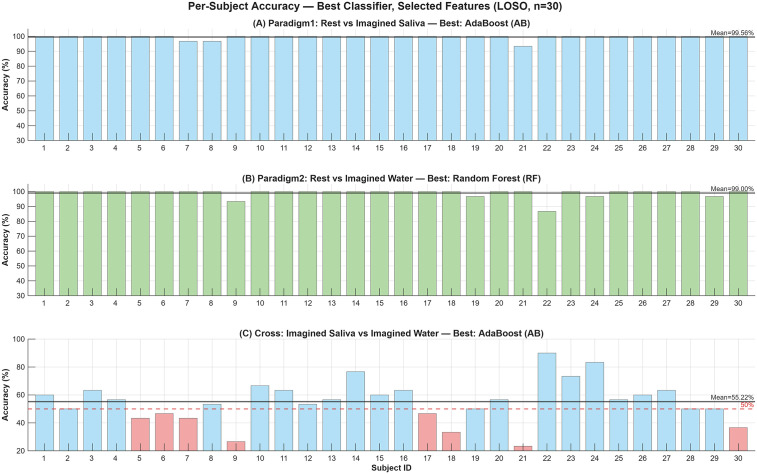
Subject-wise classification accuracies obtained using the best-performing classifiers with the selected feature set under LOSO-CV. **(A)** Paradigm 1: Classification of rest versus imagined saliva swallowing. **(B)** Paradigm 2: Classification of rest versus imagined water swallowing. **(C)** Cross-paradigm classification of imagined saliva versus imagined water swallowing.

The subject-wise classification accuracies obtained using the best-performing classifiers are presented in Fig 6 and Fig 7, corresponding to the results obtained using the full feature set and the statistically selected feature subset, respectively.

As shown in Fig 6(A) and Fig 6(B), the within-paradigm classification tasks achieved highly consistent performances across subjects. For Paradigm 1, the Support Vector Machine (SVM) classifier produced near-perfect accuracies for almost all participants, yielding a mean accuracy of 99.89%. Likewise, for Paradigm 2, the SVM classifier also demonstrated remarkably stable subject-wise performance, achieving a mean accuracy of 99.33%. Only a few participants exhibited slight reductions in accuracy, while the majority reached near-ceiling performance. These findings indicate that the extracted EEG features provide highly reliable discrimination between resting and imagined swallowing conditions within each paradigm.

In contrast, substantially greater inter-subject variability was observed for the cross-paradigm classification task shown in Fig 6(C). Although the AdaBoost (AB) classifier achieved the highest overall mean accuracy (63.56%), subject-wise performances varied considerably. Several participants achieved accuracies well above the 50% chance level, with some exceeding 90%, whereas others remained close to or below chance level. This variability highlights the increased difficulty of distinguishing between imagined saliva swallowing and imagined water swallowing, suggesting that the neural patterns associated with these two imagination tasks exhibit substantial overlap and stronger subject-dependent characteristics compared with the within-paradigm classifications.

The corresponding subject-wise results obtained using the statistically selected feature subset are illustrated in Fig 7. Similar to the full-feature analysis, near-perfect accuracies were maintained for both Paradigm 1 and Paradigm 2. For Paradigm 1, the AdaBoost classifier achieved the highest mean accuracy of 99.56%. In Paradigm 2, AdaBoost and Random Forest jointly achieved the best performance, both reaching a mean accuracy of 99.00%. These results further demonstrate that the feature selection strategy preserved the discriminative information required for within-paradigm classification.

For the cross-paradigm task shown in Fig 7(C), the overall performance remained limited despite feature selection. Although some subjects achieved accuracies substantially above chance level, considerable inter-subject variability persisted, and the highest mean accuracy obtained by AdaBoost remained relatively low (55.22%). The larger variability observed in the cross-paradigm task suggests that the neural representations of imagined saliva and imagined water swallowing exhibit substantial overlap across participants.

Overall, the subject-level analysis confirms that the proposed framework provides highly stable and consistent performance for within-paradigm imagined swallowing classification, whereas cross-paradigm decoding remains considerably more challenging due to the similarity of the underlying neural patterns.

### 3.6 Confusion matrix analysis

**Fig 8:**
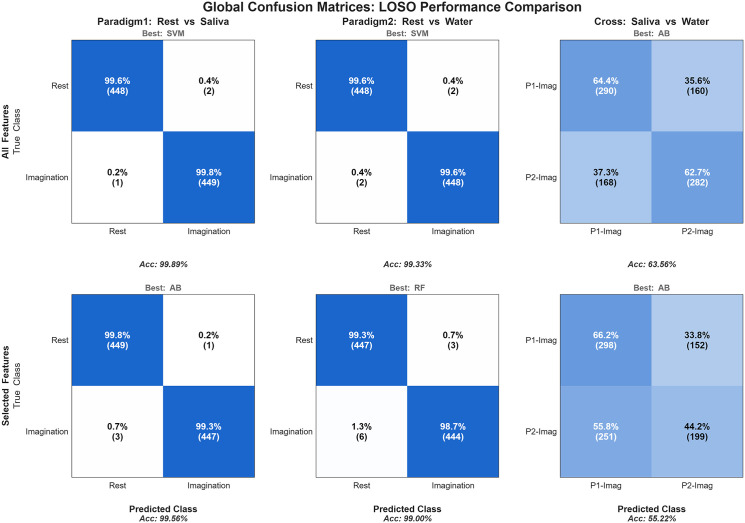
Confusion matrices of best-performing classifiers under LOSO (n = 30). Top: all features; bottom: selected features.

To further evaluate the classification performance of the best-performing models, the global confusion matrices obtained under leave-one-subject-out (LOSO) cross-validation are presented in Fig 8. The upper row illustrates the results obtained using all extracted features, whereas the lower row presents the performance achieved using the statistically selected feature subset.

For Paradigm 1 (rest vs. imagined saliva swallowing), the confusion matrices demonstrate exceptionally high classification performance for both feature configurations. Using all extracted features, the Support Vector Machine (SVM) classifier achieved an overall accuracy of 99.89%, correctly classifying 99.6% of resting samples and 99.8% of imagined swallowing samples, with only three misclassified samples in total. When the statistically selected feature subset was used, the AdaBoost (AB) classifier achieved an overall accuracy of 99.56%, correctly classifying 99.8% of resting samples and 99.3% of imagined swallowing samples. These results indicate that both the complete and reduced feature sets preserve highly discriminative information for distinguishing resting and imagined saliva swallowing states.

A similarly strong classification performance was observed for Paradigm 2 (rest vs. imagined water swallowing). Using all extracted features, the Support Vector Machine (SVM) classifier achieved an overall accuracy of 99.33%, correctly classifying 99.6% of resting samples and 99.6% of imagined swallowing samples. Using the statistically selected feature subset, the Random Forest (RF) classifier achieved an overall accuracy of 99.00%, with correct classification rates of 99.3% for resting samples and 98.7% for imagined swallowing samples. These findings further confirm the robustness and stability of the proposed feature extraction framework for within-paradigm classification tasks.

In contrast, the cross-paradigm classification task (imagined saliva swallowing vs. imagined water swallowing) presents a substantially more challenging problem. Using all extracted features, the AdaBoost classifier achieved an overall accuracy of 63.56%, correctly classifying 64.4% of imagined saliva swallowing samples and 62.7% of imagined water swallowing samples. When the selected feature subset was used, the AdaBoost classifier achieved an overall accuracy of 55.22%, with correct classification rates of 66.2% for imagined saliva swallowing samples and 44.2% for imagined water swallowing samples. Compared with the within-paradigm tasks, the increased confusion between the two imagination conditions indicates a considerable overlap in the underlying neural activation patterns associated with imagined saliva and imagined water swallowing.

Overall, the confusion matrix analysis demonstrates that the proposed EEG feature extraction and classification framework provides highly reliable performance for within-paradigm swallowing imagination tasks, achieving near-perfect discrimination between resting and motor imagery conditions. However, the comparatively lower performance observed in the cross-paradigm classification task suggests that imagined saliva and imagined water swallowing evoke highly similar cortical activity patterns, making their discrimination considerably more difficult.

### 3.7 Statistical validation of classification performance

To verify that the observed classification performances were not obtained by chance, permutation testing with 500 label shuffles was performed for all classifiers under each experimental condition. During each permutation, class labels were randomly reassigned while preserving the original feature structure, and the complete leave-one-subject-out (LOSO) classification procedure was repeated. The resulting null distributions were used to estimate chance-level performance and statistical significance.

The permutation test results obtained using the full feature set and the statistically selected feature subset are summarized in Tables X and XI, respectively. In all experiments, the chance accuracies derived from the shuffled-label distributions remained close to 50%, as expected for binary classification tasks.

#### 3.7.1 Full feature set.

As shown in Table 5, the within-paradigm classification tasks (Paradigm 1: rest vs. imagined saliva swallowing and Paradigm 2: rest vs. imagined water swallowing) achieved exceptionally high classification performances across nearly all classifiers. For Paradigm 1, the highest accuracy was obtained by SVM (99.89%), followed by AdaBoost (99.56%), Bagging (99.33%), and Random Forest (99.22%). Similarly, for Paradigm 2, SVM again achieved the highest performance (99.33%), while AdaBoost, Random Forest, and Bagging all exceeded 98% accuracy. All of these results were highly significant (p < 0.001), indicating that the observed performances were extremely unlikely to have occurred by chance.

**Table 5:** Permutation test results obtained using the full feature set.

Model	Paradigm 1 Acc (%)	Chance Acc (%)	p-value	Paradigm 2 Acc (%)	Chance Acc (%)	p-value	Cross-Paradigm Acc (%)	Chance Acc (%)	p-value
**KNN**	96.22	50.11	<0.001	95.22	49.98	<0.001	53.89	50.05	0.010
**SVM**	99.89	49.94	<0.001	99.33	49.96	<0.001	62.67	49.91	<0.001
**DT**	98.00	49.97	<0.001	98.00	49.97	<0.001	61.67	49.97	<0.001
**NB**	55.22	50.00	<0.001	57.56	49.95	<0.001	56.89	50.00	<0.001
**RF**	99.22	49.98	<0.001	98.89	50.06	<0.001	60.22	50.15	<0.001
**AB**	99.56	49.92	<0.001	99.11	49.88	<0.001	63.56	50.00	<0.001
**BAG**	99.33	49.96	<0.001	99.00	50.05	<0.001	61.78	50.01	<0.001
**KER**	48.78	49.96	0.762	50.67	50.12	0.392	51.33	49.96	0.212

In contrast, the cross-paradigm classification task proved considerably more challenging. The highest accuracy was achieved by AdaBoost (63.56%), followed by SVM (62.67%), Bagging (61.78%), and Decision Tree (61.67%). Although substantially lower than the within-paradigm results, these performances remained significantly above chance level, confirming the presence of discriminative EEG patterns capable of distinguishing imagined saliva swallowing from imagined water swallowing. The Kernel classifier did not achieve significant performance in any of the three classification scenarios.

**Fig 9:**
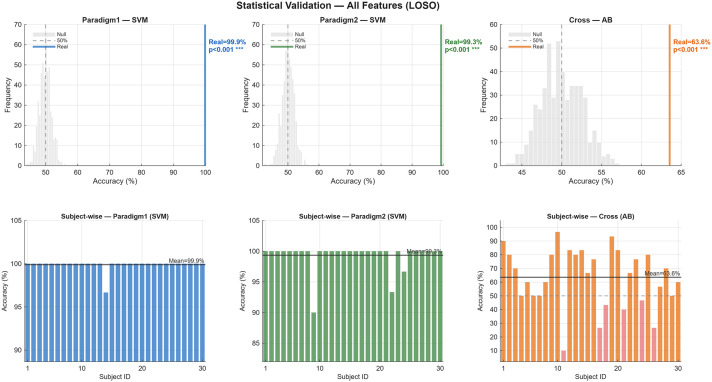
Statistical validation of classification performance using permutation testing with the full feature set. The upper panels show the null distributions generated by permutation testing, while the vertical colored line represents the real classification accuracy obtained by the best-performing classifier for each paradigm. The lower panels illustrate subject-wise classification accuracies across all participants. Results are shown for Paradigm 1, Paradigm 2, and cross-paradigm classification.

The permutation distributions corresponding to the best-performing classifiers are illustrated in Fig 9. In all cases, the observed accuracies were located well beyond the upper tails of the null distributions, resulting in statistically significant outcomes.

#### 3.7.2 Statistically selected feature subset.

The permutation test results obtained after feature selection are presented in Table 6. For the within-paradigm classification tasks, classification performance remained remarkably stable despite the substantial reduction in feature dimensionality. In Paradigm 1, AdaBoost achieved the highest accuracy (99.56%), while SVM and Random Forest both reached 99.44%. Similarly, in Paradigm 2, Random Forest and AdaBoost achieved the highest accuracy (99.00%), followed closely by SVM (98.22%). All corresponding permutation tests remained highly significant (p < 0.001), indicating that the selected features successfully preserved the most discriminative information.

**Table 6:** Permutation test results obtained using the statistically selected feature subset. *Significant at p < 0.05.

Model	Paradigm 1 Acc (%)	Chance Acc (%)	p-value	Paradigm 2 Acc (%)	Chance Acc (%)	p-value	Cross-Paradigm Acc (%)	Chance Acc (%)	p-value
**KNN**	97.44	49.96	<0.001	96.11	49.91	<0.001	50.22	49.90	0.416
**SVM**	99.44	50.02	<0.001	98.22	50.02	<0.001	51.11	49.85	0.290
**DT**	98.11	49.94	<0.001	97.89	50.00	<0.001	49.89	49.84	0.500
**NB**	67.00	49.91	<0.001	56.67	50.00	<0.001	52.89	49.86	0.044*
**RF**	99.44	50.02	<0.001	99.00	50.02	<0.001	48.78	49.99	0.746
**AB**	99.56	50.06	<0.001	99.00	50.05	<0.001	55.22	50.01	0.018*
**BAG**	99.33	49.89	<0.001	98.67	49.95	<0.001	48.67	49.91	0.774
**KER**	50.11	50.06	0.490	49.00	50.09	0.754	54.78	49.81	<0.001

A different trend was observed for the cross-paradigm classification task. Compared with the full feature set, classification performance decreased substantially for most classifiers. Only AdaBoost (55.22%, p = 0.018), Naïve Bayes (52.89%, p = 0.044), and Kernel (54.78%, p < 0.001) achieved statistically significant results, whereas the remaining classifiers produced performances close to chance level. These findings suggest that the complete feature set contains additional information required for discriminating between imagined saliva and imagined water swallowing, and that part of this information was removed during the feature selection process.

**Fig 10:**
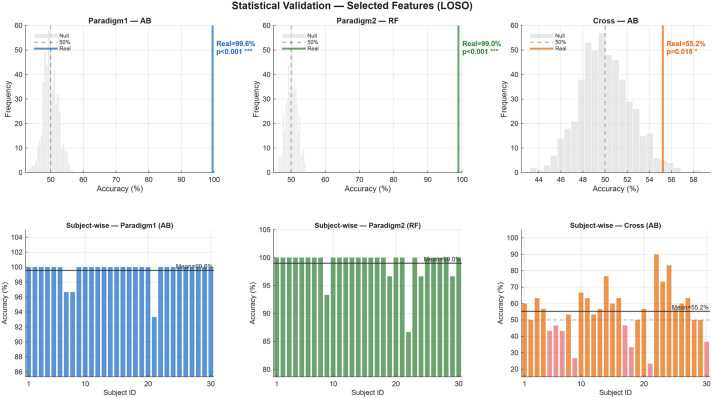
Statistical validation of classification performance using permutation testing for the statistically selected feature subset.

The corresponding permutation distributions for the selected feature subset are presented in Fig 10. Although reduced performance was observed in the cross-paradigm task, the best-performing classifiers still achieved accuracies significantly above the empirical chance distributions.

Overall, the permutation analyses confirm that the exceptionally high accuracies obtained in both within-paradigm classification tasks are not attributable to random chance. Furthermore, despite the lower accuracies observed in the cross-paradigm task, the statistically significant results indicate the existence of partially shared yet distinguishable neural representations associated with imagined saliva swallowing and imagined water swallowing.

Importantly, all models were evaluated using LOSO cross-validation, ensuring subject-independent assessment. Under this rigorous evaluation framework, the consistently high accuracies and significant permutation test outcomes demonstrate that the extracted EEG features capture stable and generalizable neural patterns associated with imagined swallowing. These findings provide strong evidence for the robustness and reliability of the proposed EEG-based classification framework.

### 3.8 Temporal cue analysis

**Fig 11:**
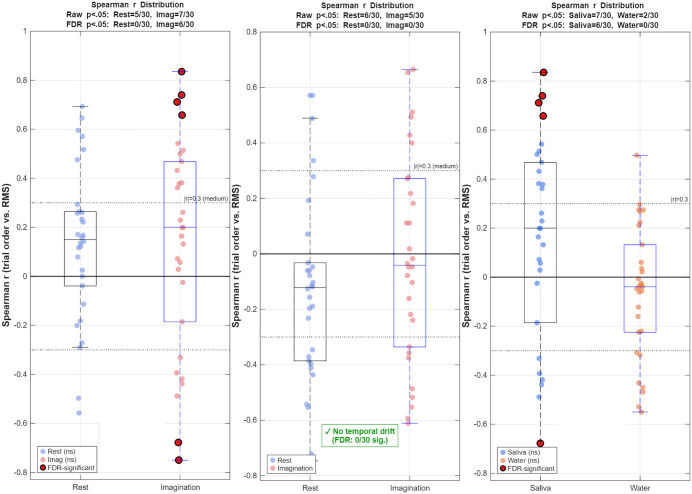
Temporal cue analysis across all experimental conditions.

To rule out the possibility that classification performance was driven by systematic temporal drift in EEG amplitude across trials, we conducted a temporal cue analysis for all three experimental conditions (see Fig 11). For each subject, the Spearman rank correlation between trial order (1–15) and epoch-level RMS amplitude was computed separately for each epoch type. To correct for multiple comparisons across 30 subjects, the Benjamini–Hochberg FDR procedure was applied independently for each epoch condition (α = 0.05).

For Paradigm 1, no significant trial-order trend was observed in the rest epoch after FDR correction (mean r = 0.133 ± 0.316; FDR-corrected: 0/30 subjects; Fig 11, left panel). In the imagination epoch, 6 out of 30 subjects showed a significant positive trend (mean r = 0.138 ± 0.433; FDR-corrected: 6/30). This pattern was asymmetric between conditions (rest: 0/30 vs. imagination: 6/30) and consistently positive in direction, which is inconsistent with a classifiable temporal confound. A classifiable artifact would be expected to affect both conditions symmetrically; the observed pattern is instead consistent with within-session motor imagery skill consolidation, a well-documented neurophysiological phenomenon in which cortical engagement increases across repeated trials as participants become more proficient at the task.

For Paradigm 2, no significant trend was observed after FDR correction in either condition (rest: mean r = −0.130 ± 0.344, FDR-corrected: 0/30; imagination: mean r = −0.015 ± 0.373, FDR-corrected: 0/30; Fig 11, center panel). The uncorrected significant findings (rest: 6/30; imagination: 5/30) are consistent with the number of false positives expected by chance under 30 simultaneous comparisons (expected ≈ 1.5).

For the cross-paradigm condition, no significant trend was observed in the water imagination epoch (mean r = −0.060 ± 0.270; FDR-corrected: 0/30), while 6 out of 30 subjects showed a significant positive trend in the saliva imagination epoch (mean r = 0.138 ± 0.433; FDR-corrected: 6/30; Fig 11, right panel), mirroring the Paradigm 1 imagination finding and attributable to the same within-session consolidation effect. Furthermore, the LOSO cross-validation framework provides a structural safeguard against temporal confounds: since each test subject’s trials are evaluated as a complete block encompassing all trial positions, any within-subject temporal drift affects all positions equally and cannot systematically favor either class label. Taken together, these results confirm that the classification accuracies reported across all three experimental conditions are not attributable to trial-order temporal cues or amplitude drift artifacts.

### 3.9 Feature analysis and spatial distribution

**Fig 12:**
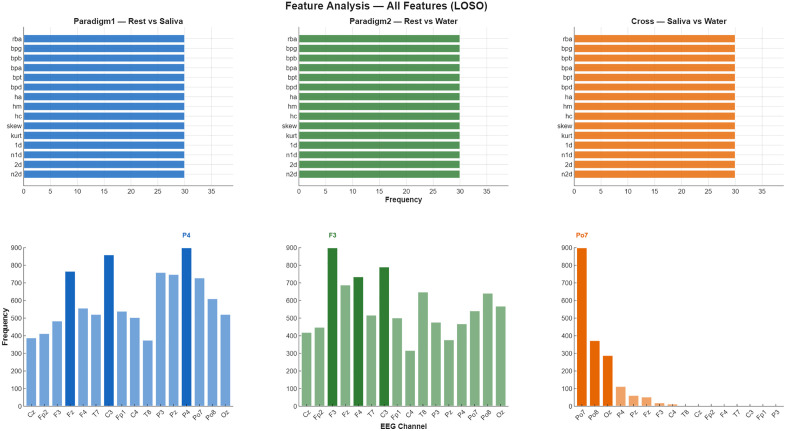
Feature and channel distribution obtained using the full feature set. The upper panels show the frequency of feature types across LOSO folds for Paradigm 1, Paradigm 2, and cross-paradigm classification. The lower panels illustrate the spatial distribution of EEG channels based on feature occurrence frequency.

**Fig 13:**
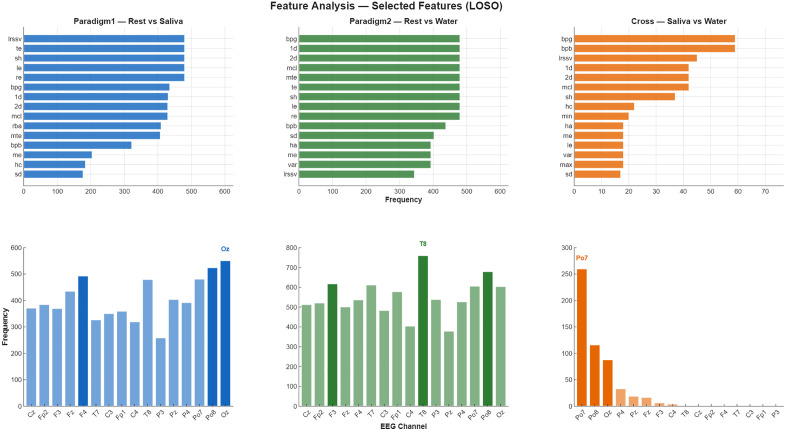
Feature analysis using the statistically selected feature subset. The upper panels display the most frequently selected discriminative features across LOSO folds for Paradigm 1, Paradigm 2, and cross-paradigm classification. The lower panels show the spatial distribution of EEG channels associated with the selected features.

To further investigate which EEG characteristics contributed to the classification performance, a feature and channel distribution analysis was conducted. The results obtained using the full feature set and the statistically selected feature subset are presented in Fig 12 and Fig 13, respectively.

Figure 12 illustrates the distribution of feature types and EEG channels when the full feature set was considered. The upper panels show the frequency of different feature categories across LOSO folds for each paradigm. The feature-type distributions were relatively uniform in Paradigm 1, Paradigm 2, and the cross-paradigm task, indicating that discriminative information was broadly distributed across spectral, statistical, and Hjorth-based descriptors rather than being dominated by a small subset of features.

The spatial distribution of EEG channels, shown in the lower panels of Fig 12, revealed distinct patterns across the three classification tasks. In Paradigm 1 (rest vs. imagined saliva swallowing), higher feature frequencies were predominantly observed over parietal regions, with P4 exhibiting the strongest contribution, followed by notable involvement of frontal and temporal electrodes. In Paradigm 2 (rest vs. imagined water swallowing), F3 showed the highest occurrence frequency, although substantial contributions were also observed in central and parietal regions, suggesting that task-related information was distributed across multiple scalp regions. In contrast, the cross-paradigm classification task (imagined saliva vs. imagined water swallowing) exhibited a highly localized pattern, with PO7 demonstrating an overwhelmingly dominant contribution compared with all other electrodes. This finding suggests that the neural differences between imagined saliva and imagined water swallowing may be represented by more spatially localized EEG activity patterns.

While Fig 12 reflects the contribution of the complete feature set, Fig 13 presents the distributions obtained after statistical feature selection, enabling the identification of the most stable and consistently informative EEG characteristics across LOSO folds. Compared with the full feature set, a smaller and more consistent subset of discriminative features emerged following feature selection. Several statistical and spectral descriptors, including Hjorth-derived measures, variance-related features, RMS-based measures, and β-band power features, were repeatedly selected in both Paradigm 1 and Paradigm 2, indicating that these features contain stable discriminative information across subjects.

The spatial distribution of the selected channels further revealed paradigm-specific patterns. In Paradigm 1, the Oz channel exhibited the highest occurrence frequency among the selected features, suggesting a stronger contribution of posterior scalp regions to the discrimination of imagined saliva swallowing from rest. In Paradigm 2, the T8 channel showed the strongest contribution, indicating a greater contribution of temporal-region EEG activity in distinguishing imagined water swallowing from rest. For cross-paradigm classification, discriminative features were highly concentrated around the PO7 channel, suggesting that the neural differences between imagined saliva and imagined water swallowing are relatively subtle and spatially localized.

Taken together, the analyses of both the full feature set and the statistically selected feature subset demonstrate that imagined swallowing tasks are characterized by distributed EEG activity patterns involving multiple scalp regions. While the complete feature set revealed broadly distributed discriminative information, statistical feature selection highlighted a smaller set of stable and repeatedly selected descriptors. Both spectral and statistical EEG features contributed to classification performance. Furthermore, the consistency of the selected features and channels across LOSO folds supports the robustness of the feature selection procedure and the subject-independent generalizability of the proposed framework.

## 4. Discussion

This study unveils the potential of EEG-based features in understanding and differentiating swallowing-related brain signals. The identified features demonstrate high accuracy in classifying different swallowing states during induced saliva and water swallowing experiments. These findings provide methodological evidence for the feasibility of decoding imagined swallowing intention under controlled laboratory conditions. However, it is important to emphasize that the present study was conducted exclusively on healthy participants, and therefore the findings cannot be directly generalized to clinical populations with dysphagia.

While the results are promising from a signal decoding perspective, they should be interpreted as a proof-of-concept rather than evidence of clinical applicability. Accordingly, this study was not designed to evaluate diagnostic performance, therapeutic outcomes, or comparisons with established swallowing assessment techniques.

In the established clinical pathway, dysphagia assessment and rehabilitation are led by Speech-Language Pathologists (SLPs), who administer gold-standard instrumental procedures such as Videofluoroscopic Swallowing Study (VFSS) and Fiberoptic Endoscopic Evaluation of Swallowing (FEES) to characterize swallowing physiology and guide treatment decisions [8,9]. The present EEG-based framework is not intended to replace these procedures, but rather to serve as a complementary, non-invasive tool that may support SLP-led workflows—for instance, by enabling repeated monitoring of cortical engagement during rehabilitation or by providing objective indices of motor imagery training progress that are not captured by instrumental swallowing assessments alone.

Overall, the findings of this study highlight three main contributions. First, the results demonstrate the feasibility of reliably distinguishing rest from imagined swallowing using EEG-based features across both saliva and water swallowing paradigms. Second, the study provides evidence for partial but meaningful discrimination between saliva and water imagined swallowing, indicating that EEG signals capture subtle differences between closely related motor imagery tasks. Finally, these findings establish a technical foundation that may inform future interdisciplinary investigations into EEG-based motor imagery paradigms.

Previous electrophysiological studies have demonstrated that swallowing involves complex cortical integration, supporting the relevance of EEG-based approaches [19,20]. To further contextualize the present findings within the existing literature, a comparative overview of prior motor imagery (MI) swallowing studies is provided in Table 7. Earlier EEG-based investigations, particularly those conducted by Yang et al. [17,34], primarily focused on distinguishing imagined swallowing (MI-SW) from rest or tongue motor imagery tasks, often within single-session or cross-session frameworks. Reported classification accuracies in healthy individuals generally ranged between approximately 69% and 74%, while stroke populations demonstrated slightly lower performance levels (66–70%). These studies emphasized feature selection strategies and model adaptation techniques to improve cross-session robustness [21,34,35,36].

**Table 7:** Motor ımagery in swallowing research: summary of key studies.

Author(s) (Year)	Method	Objective	Key Findings
Yang et al. (2012)	EEG	MI-SW detection for stroke rehabilitation	Time-segment selection based on tongue movement improved accuracy (69.96%).
Yang et al. (2013a,b)	EEG	MI-SW and MI-TM classification; model adaptation	Session accuracy: ~72–74%; Feature consistency and cluster impurity-based model adaptation improved cross-session performance.
Yang et al. (2014)	EEG	Hemodynamics during MI and ME of swallowing/tongue	Accuracy: healthy ~71–74%, stroke ~66–70%; MI differed significantly from the rest.
Yang et al. (2016)	EEG	Correlation among MI-SW, MI-TM, and ME-SW	Strong correlations at C3 in mu/low-beta bands; weaker in stroke group.
Kober & Wood (2014)	NIRS	Brain response to MI and ME of swallowing	Frontal and premotor activations; desoxy-Hb correlated with MI and ME.
Kober et al. (2015a)	NIRS	Compare MI and ME in dysphagic vs. healthy	The dysphagic group showed stronger frontal hemodynamic changes.
Kober et al. (2015b)	NIRS	NIRS-based neurofeedback effects on swallowing	Desoxy-Hb training increased frontal activation; no effect in the oxy-Hb group.

Additionally, previous NIRS-based investigations highlighted frontal and premotor activations during both motor imagery and motor execution of swallowing, including differential hemodynamic responses in dysphagic versus healthy populations. While these studies primarily examined cortical activation patterns and neurofeedback effects, they did not extensively address fine-grained electrophysiological discrimination between closely related imagined swallowing contexts [37–39].

By extending prior binary paradigms to include differentiation between imagined saliva and imagined water swallowing, the present work contributes to a more nuanced understanding of swallowing-related motor imagery. Rather than focusing solely on rest-versus-imagery detection, this study explores contextual variability within swallowing imagery, thereby addressing a gap identified in earlier literature.

In contrast, the present study achieved higher classification performance for rest versus imagined swallowing under a subject-independent validation framework. It is important to note, however, that methodological differences—including experimental documentation, validation strategy, participant characteristics, and feature extraction approaches—limit direct numerical comparison. Nevertheless, the improved decoding performance observed in the current study may be attributed to the comprehensive feature engineering strategy and ensemble-based classification methods employed.

While these higher accuracies may appear substantially above typical motor imagery studies, this difference can be explained by the use of a subject-independent LOSO framework, fold-wise feature selection, and permutation-based statistical validation, which collectively reduce the risk of data leakage and support the reliability of the reported results.

The observed electrophysiological differences between imagined saliva and water swallowing suggest that EEG-derived features are sensitive to contextual variations in motor imagery planning. This finding is consistent with the broader literature linking motor imagery to motor system engagement during rehabilitation research. In this context, the present study aimed to examine whether distinct imagined swallowing contexts could be differentiated electrophysiologically in a subject-independent framework.

Although motor imagery exercise cannot replace active exercise in general, it is recommended to use motor imagery to increase active exercise in motor learning and motor performance, especially after neurological damage [40–42]. The most effective approach is attained when physiotherapy is combined with motor imagery [40–43]. Moreover, studies in the literature have explored different methodologies of swallow motor imagery [34–44], including recent deep learning-based approaches [45,46].

Our findings align with this evidence, indicating that imagined swallowing activates specific brain regions and feature patterns, even in the absence of overt motion. Although our study did not include actual swallowing, the differential EEG activity observed during imagination confirms that intention-related cortical dynamics can be captured without overt movement.

A comprehensive comparison of eight machine learning classifiers showed that ensemble-based methods (Random Forest, AdaBoost, and Bagging), together with SVM, consistently achieved the highest classification performance. For rest versus imagined swallowing, classification accuracies reached approximately 99% across both paradigms under LOSO validation. Importantly, these models maintained comparable performance when only statistically selected features were used, indicating the robustness and stability of the extracted feature set.

These results demonstrate the feasibility of reliably distinguishing rest from imagined swallowing, which represents a fundamental requirement for EEG-based swallowing intention decoding. Importantly, the clear performance gap between within-paradigm and cross-paradigm classification suggests that the model captures task-specific neural representations rather than relying on trivial or non-neural cues.

In contrast, classifiers such as Naive Bayes and Kernel-based methods showed substantially lower performance, suggesting that simpler probabilistic or non-optimized models are insufficient to capture the nonlinear and distributed characteristics of swallowing-related EEG patterns.

Although rest versus imagination classification yielded very high performance, differentiating between imagined saliva and imagined water swallowing remained more challenging. The highest accuracy reached approximately 60%, with a consistent reduction in performance across classifiers. This moderate performance likely reflects the substantial overlap in neural representations between these closely related motor imagery tasks rather than a limitation of the proposed framework. It is important to clarify that the saliva versus water imagined swallowing comparison was not conceived as a clinically operational classification task. Rather, it was designed to address a fundamental neurophysiological question: do these two contextually distinct swallowing imagery conditions—which differ in oral sensory input, bolus volume, preparatory motor planning, and degree of volitional control—engage measurably different cortical dynamics despite their largely overlapping neural pathways? In this sense, the approximately 60% above-chance, permutation-validated accuracy constitutes genuine evidence of cortical separability between two highly similar imagined conditions, not a failure of discrimination. The clinical significance of this finding lies not in its immediate translational application, but in its demonstration that cortical swallowing representations retain sensitivity to subtle contextual differences during motor imagery—a property that future, more targeted paradigms may exploit in the design of context-aware neurofeedback systems.

Importantly, achieving above-chance discrimination between such similar conditions indicates that EEG-derived features are sensitive to subtle variations in swallowing-related cortical activity. While these results may appear unusually strong, the combination of LOSO validation, fold-wise processing, and permutation testing supports that the performance is not driven by data leakage or overfitting.

At present, however, the study does not establish clinical efficacy, and no therapeutic claims are made. Future work must include dysphagic populations, interdisciplinary clinical expertise, and validation against established assessment methods before translational conclusions can be drawn.

Our findings contribute to the methodological understanding of EEG-based imagined swallowing analysis. However, several limitations must be addressed to ensure the robustness and clinical applicability of these results.

In this study, we intentionally excluded comparisons involving actual swallowing to minimize the influence of motion artifacts and electromyographic interference. It is worth noting that in BCI-based rehabilitation paradigms, the motor imagery signal is typically decoded prior to overt movement initiation rather than during it. In this context, imagined swallowing serves as a cortical intention signal that could potentially trigger neurofeedback or assistive responses before the swallowing act begins—an approach analogous to upper-limb BCI systems where imagined movement precedes attempted execution. While this approach enhances signal reliability for BCI applications, future studies incorporating actual swallowing with advanced artifact reduction techniques may provide deeper insights.

The study was conducted on healthy participants under controlled laboratory conditions, and future work should include dysphagic populations and real-time feedback systems to validate clinical effectiveness. Additionally, future translational studies should be co-designed with SLPs, who possess the clinical expertise to determine which patient populations, assessment contexts, and rehabilitation goals would most benefit from EEG-based motor imagery monitoring. Integration of EEG-derived indices into SLP-led treatment plans—alongside existing outcome measures such as VFSS or FEES findings, dietary level progression, and standardized swallowing scales—will be essential to establish the clinical relevance of this approach [8–10]. Future studies should consider this direction, provided that appropriate noise reduction or multimodal signal fusion techniques are applied to preserve signal quality.

Additional limitations include the modest sample size and limited demographic diversity, which may not fully represent clinical populations. Moreover, the use of a subject-independent leave-one-subject-out (LOSO) validation framework allowed us to explicitly evaluate generalizability across individuals, thereby addressing inter-subject variability inherent to EEG data. Moreover, experimental swallowing scenarios may not fully capture the complexity of natural or spontaneous swallowing behavior. EEG, although non-invasive and temporally precise, is also prone to noise, which can affect feature extraction and classification accuracy.

Inter-individual variability in performing motor imagery tasks and the complexity of interpreting EEG patterns further complicate generalization. The LOSO validation strategy partially mitigates this concern by enforcing cross-subject generalization. Translating these insights into functional BCI-based rehabilitation tools will require overcoming technological and logistical challenges, including interface usability, signal robustness in real-world settings, and long-term efficacy. Comparative studies against conventional therapy methods are essential to determine the true therapeutic value of EEG-based approaches.

Despite these challenges, our study aligns with the broader scope of neurological research utilizing EEG to analyze brain function. The method’s cost-effectiveness, portability, and high temporal resolution make it well-suited for investigating dynamic processes such as swallowing. Whether such benefits can be realized clinically remains to be determined through controlled patient studies.

Looking forward, expanding the research to include diverse swallowing types, demographic groups, and real-time feedback applications will be critical. In particular, such future developments require rigorous clinical validation before practical implementation can be considered. Crucially, this translational pathway must be grounded in existing clinical structures: SLPs, as the primary clinicians responsible for dysphagia management, should be central collaborators in the design, validation, and implementation of any EEG-based tool intended for swallowing rehabilitation [7].

## 5. Conclusion

This study investigated the electrophysiological signatures of imagined swallowing by analyzing EEG-based features during motor imagery of two different tasks: saliva and water swallowing. Through rigorous preprocessing, statistical testing, and feature engineering, we identified a subset of EEG-derived features—especially entropy-based and energy-related measures—that showed robust differences between rest and imagined swallowing conditions. Using a range of machine learning classifiers, the system achieved high classification accuracy (up to 99%) for distinguishing rest from imagined swallowing and moderate success (up to 63%) in separating imagined saliva from water swallowing.

Among the classification algorithms, ensemble methods—Random Forest, AdaBoost, and Bagging—together with SVM consistently outperformed others, demonstrating their capacity to model the non-linear and distributed nature of EEG motor imagery data.

Importantly, while our findings reveal that imagined swallowing evokes measurable and distinct cortical responses, we acknowledge that this study was limited to healthy individuals and did not include actual swallowing data. Therefore, conclusions regarding clinical implementation should be interpreted with caution. The present study was not designed to evaluate diagnostic accuracy, therapeutic efficacy, or comparative effectiveness against established swallowing assessment methods. Instead, the findings provide a proof-of-concept framework for future methodological investigations.

Furthermore, the use of subject-independent LOSO validation, fold-wise feature selection, and permutation testing supports that the reported performance is not driven by data leakage or overfitting, but reflects stable and generalizable neural patterns.

Future work should include patient populations, utilize source localization techniques to explore involved brain regions, and incorporate real-time neurofeedback. Any potential translational or therapeutic application will require rigorous clinical validation, interdisciplinary collaboration, and comparison with established clinical assessment techniques.

In summary, this study contributes to the expanding literature on motor imagery for swallowing by demonstrating the robustness of decoding imagined swallowing states using EEG. The results highlight the potential of EEG-based approaches for capturing subtle variations in swallowing-related motor imagery under controlled conditions. While further validation is essential, these findings represent a methodological step toward understanding the neural dynamics of imagined swallowing rather than a clinically deployable solution.

## References

[1] Yang S, Park JW, Min K, Lee YS, Song YJ, Choi SH, et al. Clinical practice guidelines for oropharyngeal dysphagia. Ann Rehabil Med. 2023;47(Suppl 1):S1–26. doi: 10.5535/arm.23069 PMC10405672 37501570

[2] Dudik JM, Kurosu A, Coyle JL, Sejdić E. Dysphagia and its effects on swallowing sounds and vibrations in adults. Biomed Eng Online. 2018;17(1):1–18. doi: 10.1186/s12938-018-0501-9 29855309 PMC5984479

[3] Bath 3 P M, Lee HS, Everton LF. Swallowing therapy for dysphagia in acute and subacute stroke. Cochrane Database Syst Rev. 2018;2018(10). doi: 10.1002/14651858.CD000323.pub3 PMC6516809 30376602

[4] Leslie P, Drinnan MJ, Zammit-Maempel I, Coyle JL, Ford GA, Wilson JA. Cervical auscultation synchronized with images from endoscopy swallow evaluations. Dysphagia. 2007;22(4):290–8. doi: 10.1007/s00455-007-9084-5 17554472

[5] Langmore SE. Evaluation of oropharyngeal dysphagia: which diagnostic tool is superior? Curr Opin Otolaryngol Head Neck Surg. 2003;11(6):485–9. doi: 10.1097/00020840-200312000-00014 14631184

[6] Coyle JL, Davis LA, Easterling C, Graner DE, Langmore S, Leder SB, et al. Oropharyngeal dysphagia assessment and treatment efficacy: setting the record straight (response to Campbell-Taylor). J Am Med Dir Assoc. 2009;10(1):62–6; discussion 79-83. doi: 10.1016/j.jamda.2008.10.003 19111855

[7] American Speech-Language-Hearing Association (ASHA). Speech-language pathologists as the preferred providers for dysphagia services. Available from: https://www.asha.org/slp/healthcare/speech-language-pathologists-as-the-preferred-providers-for-dysphagia-services/

[8] Chiu Y-H. The Roles of Videofluoroscopic Swallowing Study (VFSS) and Fiberoptic Endoscopic Evaluation of Swallowing (FEES) in the assessment of dysphagia. RPS. 2020;48(2). doi: 10.6315/tjpmr.202012_48(2).0001

[9] Langmore SE. Endoscopic evaluation and treatment of swallowing disorders. New York, NY, USA: Thieme; 2001.

[10] Logemann J. Evaluation and treatment of swallowing disorders. NSSLHA Journal. 1984;12:38–50.

[11] Binnie CD, Prior PF. Electroencephalography. J Neurol Neurosurg Psychiatry. 1994;57(11):1308–19. doi: 10.1136/JNNP.57.11.1308 7964803 PMC1073178

[12] Attwell D, Laughlin SB. Journal of cerebral blood flow and metabolism: official journal of the International Society of Cerebral Blood Flow and Metabolism. J Cereb Blood Flow Metab. 2001;21(10):1133–45. doi: 10.1097/00004647-200110000-00001 11598490

[13] Muehllehner G, Karp JS. Positron emission tomography. Phys Med Biol. 2006;51(13):R117. doi: 10.1088/0031-9155/51/13/R08 16790899

[14] Proudfoot M, Woolrich MW, Nobre AC, Turner MR. Magnetoencephalography. Pract Neurol. 2014;14(5):336–43. doi: 10.1136/PRACTNEUROL-2013-000768 24647614 PMC4174130

[15] Jordan KG. Continuous EEG and evoked potential monitoring in the neuroscience intensive care unit. J Clin Neurophysiol. 1993;10(4):445–75. doi: 10.1097/00004691-199310000-00006 8308143

[16] Drake W, O’Donoghue S, Bartram C, Lindsay J, Greenwood R. Eating in side-lying facilitates rehabilitation in neurogenic dysphagia. Brain Inj. 1997;11(2):137–42. doi: 10.1080/026990597123737 9012947

[17] Huijuan Yang, Guan C, Ang KK, Wang CC, Phua KS, Juanhong Yu. Dynamic initiation and dual-tree complex wavelet feature-based classification of motor imagery of swallow EEG signals. In: The 2012 International Joint Conference on Neural Networks (IJCNN). 2012. pp. 1–6. 10.1109/ijcnn.2012.6252603

[18] Jestrović I, Coyle JL, Perera S, Sejdić E. Influence of attention and bolus volume on brain organization during swallowing. Brain Struct Funct. 2018;223(2):955–64. doi: 10.1007/S00429-017-1535-7 29058086

[19] Huckabee M-L, Deecke L, Cannito MP, Gould HJ, Mayr W. Cortical control mechanisms in volitional swallowing: the Bereitschaftspotential. Brain Topogr. 2003;16(1):3–17. doi: 10.1023/a:1025671914949 14587965

[20] Jestrović I, Coyle JL, Sejdić E. Decoding human swallowing via electroencephalography: a state-of-the-art review. J Neural Eng. 2015;12(5):051001. doi: 10.1088/1741-2560/12/5/051001 26372528 PMC4596245

[21] Yang H, Ang KK, Wang C, Phua KS, Guan C. Neural and cortical analysis of swallowing and detection of motor imagery of swallow for dysphagia rehabilitation-a review. Prog Brain Res. 2016;228:185–219. doi: 10.1016/bs.pbr.2016.03.014 27590970

[22] Kober SE, Grössinger D, Wood G. Effects of motor imagery and visual neurofeedback on activation in the swallowing network: a real-time fMRI study. Dysphagia. 2019;34(6):879–95. doi: 10.1007/s00455-019-09985-w 30771088 PMC6825652

[23] Aslan SG, Yılmaz B. Examining tongue movement intentions in EEG-based BCI with machine and deep learning: an approach for dysphagia rehabilitation. EuroBiotech J. 2024;8(4):176–83. doi: 10.2478/ebtj-2024-0017

[24] Aslan SG, Yilmaz B. Distinguishing resting state from motor imagery swallowing using EEG and deep learning models. IEEE Access. 2024;12:178375–89. doi: 10.1109/ACCESS.2024.3501013

[25] Zheng L, et al. Ensemble learning method based on temporal, spatial features with multi-scale filter banks for motor imagery EEG classification. Biomed Signal Process Control. 2022;76. doi: 10.1016/j.bspc.2022.103634

[26] Yu Z, Chen W, Zhang T. Motor imagery EEG classification algorithm based on improved lightweight feature fusion network. Biomed Signal Process Control. 2022;75. doi: 10.1016/j.bspc.2022.103618

[27] Zhao J, Liu M. A deep temporal network for motor imagery classification based on multi-branch feature fusion and attention mechanism. Biomed Signal Process Control. 2025;100. doi: 10.1016/j.bspc.2024.107163

[28] Gtech. Nautilus research wearable EEG headset. Available from: https://www.gtec.at/wp-content/uploads/2019/09/g.Nautilus-research-wearable-eeg-headset.jpg. 2019.

[29] Delorme A, Makeig S. EEGLAB: an open source toolbox for analysis of single-trial EEG dynamics including independent component analysis. J Neurosci Methods. 2004;134(1):9–21. doi: 10.1016/j.jneumeth.2003.10.009 15102499

[30] Nawaz R, Cheah KH, Nisar H, Yap VV. Comparison of different feature extraction methods for EEG-based emotion recognition. Biocybern Biomed Eng. 2020;40(3). doi: 10.1016/j.bbe.2020.04.005

[31] Jenke R, Peer A, Buss M. Feature extraction and selection for emotion recognition from EEG. IEEE Trans Affective Comput. 2014;5(3):327–39. doi: 10.1109/taffc.2014.2339834

[32] Kaleem MF, Sugavaneswaran L, Guergachi A, Krishnan S. Application of empirical mode decomposition and Teager energy operator to EEG signals for mental task classification. Annu Int Conf IEEE Eng Med Biol Soc. 2010;2010:4590–3. doi: 10.1109/IEMBS.2010.5626501 21096224

[33] Janifer Jabin Jui S, Deo RC, Barua PD, Devi A, Soar J, et al. Application of entropy for automated detection of neurological disorders with electroencephalogram signals: a review of the last decade (2012-2022). 2023. IEEE. 10.1109/ACCESS.2023.3294473

[34] Yang H, Guan C, Ang KK, Wang C, Phua KS, Yin CTK, et al. Feature consistency-based model adaptation in session-to-session classification: a study using motor imagery of swallow EEG signals. Annu Int Conf IEEE Eng Med Biol Soc. 2013;2013:429–32. doi: 10.1109/EMBC.2013.6609528 24109715

[35] Yang H, Guan C, Chua KSG, Chok SS, Wang CC, Soon PK, et al. Detection of motor imagery of swallow EEG signals based on the dual-tree complex wavelet transform and adaptive model selection. J Neural Eng. 2014;11(3):035016. doi: 10.1088/1741-2560/11/3/035016 24836742

[36] Yang H, Guan C, Wang CC, Ang KK, Phua KS, Chok SS, et al. On the correlations of motor imagery of swallow with motor imagery of tongue movements and actual swallow. In: Advances in cognitive neurodynamics. Springer Singapore; 2016. p. 397–404. 10.1007/978-981-10-0207-6_55

[37] Kober SE, Wood G. Changes in hemodynamic signals accompanying motor imagery and motor execution of swallowing: a near-infrared spectroscopy study. Neuroimage. 2014;93 Pt 1:1–10. doi: 10.1016/j.neuroimage.2014.02.019 24576696

[38] Kober SE, Bauernfeind G, Woller C, Sampl M, Grieshofer P, Neuper C, et al. Hemodynamic signal changes accompanying execution and imagery of swallowing in patients with dysphagia: a multiple single-case near-infrared spectroscopy study. Front Neurol. 2015;6:151. doi: 10.3389/fneur.2015.00151 26217298 PMC4491622

[39] Kober SE, Gressenberger B, Kurzmann J, Neuper C, Wood G. Voluntary modulation of hemodynamic responses in swallowing related motor areas: a near-infrared spectroscopy-based neurofeedback study. PLoS One. 2015;10(11):e0143314. doi: 10.1371/journal.pone.0143314 26575032 PMC4648579

[40] Dickstein R, Deutsch JE. Motor imagery in physical therapist practice. Phys Ther. 2007;87(7):942–53. doi: 10.2522/PTJ.20060331 17472948

[41] Peters HT, Page SJ. Integrating mental practice with task-specific training and behavioral supports in poststroke rehabilitation: evidence, components, and augmentative opportunities. Phys Med Rehabil Clin N Am. 2015;26(4):715–27. doi: 10.1016/J.PMR.2015.06.004 26522908

[42] Feltz DL, Landers DM. The effects of mental practice on motor skill learning and performance: a meta-analysis. J Sport Psychol. 1983;5(1):25–57. doi: 10.1123/jsp.5.1.25

[43] Page SJ, Harnish S. Thinking about better speech: mental practice for stroke-induced motor speech impairments. Aphasiology. 2011;26(2):127–42. doi: 10.1080/02687038.2011.636027 PMC3269777 22308050

[44] Yang H, Guan C, Ang KK, Wang C. Detection of motor imagery of swallow with model adaptation: swallow or tongue. In: Abstract in Fifth Int. Brain Computer Interface Meeting. Vol. 56. 2013.

[45] Aslan SG, Yılmaz B. EEG-based decoding of swallowing intention using a transformer-enhanced deep learning approach. Biomed Signal Process Control. 2026;119:109861. doi: 10.1016/j.bspc.2026.109861

[46] Gökçe Aslan S, Yılmaz B. Multi-scale spatial attention network for rest and imagination classification in saliva and water swallowing paradigms. Biomed Signal Process Control. 2026;119:109966. doi: 10.1016/j.bspc.2026.109966

